# LRP1-mediated pH-sensitive polymersomes facilitate combination therapy of glioblastoma in vitro and in vivo

**DOI:** 10.1186/s12951-020-00751-x

**Published:** 2021-01-22

**Authors:** Chen He, Zhiyuan Zhang, Yinan Ding, Kangli Xue, Xihui Wang, Rui Yang, Yanli An, Dongfang Liu, Chunmei Hu, Qiusha Tang

**Affiliations:** 1 Medical School of Southeast University, 87 Dingjiaqiao Road, Nanjing, China; 2 Department of Neurosurgery, Nanjing Jinling Hospital, Nanjing University, Nanjing, China; 3 Research Institute for Reproductive Health and Genetic Diseases, The Affiliated Wuxi Maternity and Child Health Care Hospital of Nanjing Medical University, Wuxi, China; 4 Department of Tuberculosis, the Second Affiliated Hospital of Southeast University, Nanjing, China

**Keywords:** pH-sensitive, Polymersomes, Glioblastoma, Brain-targeting, Combination therapy

## Abstract

**Background:**

Glioblastoma (GBM) is the most invasive primary intracranial tumor, and its effective treatment is one of the most daunting challenges in oncology. The blood–brain barrier (BBB) is the main obstacle that prevents the delivery of potentially active therapeutic compounds. In this study, a new type of pH-sensitive polymersomes has been designed for glioblastoma therapy to achieve a combination of radiotherapy and chemotherapy for U87-MG human glioblastoma xenografts in nude mice and significantly increased survival time.

**Results:**

The Au-DOX@PO-ANG has a good ability to cross the blood–brain barrier and target tumors. This delivery system has pH-sensitivity and the ability to respond to the tumor microenvironment. Gold nanoparticles and doxorubicin are designed as a complex drug. This type of complex drug improve the radiotherapy (RT) effect of glioblastoma. The mice treated with Au-DOX@PO-ANG NPs have a significant reduction in tumor volume.

**Conclusion:**

In summary, a new pH-sensitive drug delivery system was fabricated for the treatment of glioblastoma. The new BBB-traversing drug delivery system potentially represents a novel approach to improve the effects of the treatment of intracranial tumors and provides hope for glioblastoma treatment. 
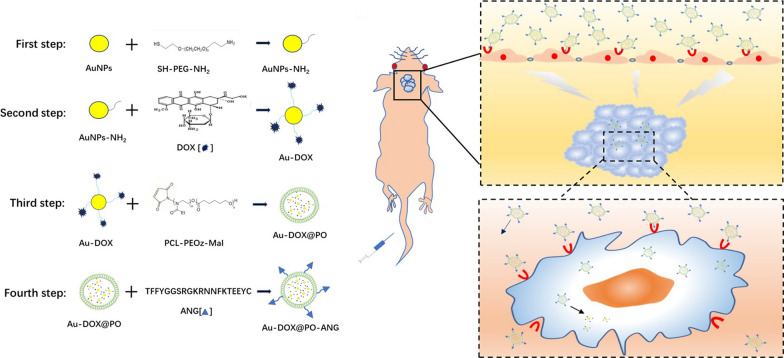

## Introduction

Glioblastoma (GBM) is the most invasive primary intracranial tumor [1–3], and its effective treatment is one of the most daunting challenges in oncology [4]. Current clinical chemotherapeutic treatments still show very limited therapeutic efficacy [5, 6].The blood–brain barrier (BBB) is the main obstacle that prevents the delivery of potentially active therapeutic compounds [7]. Moreover, limited tumor cell drug uptake and tumor resistance to chemotherapy have been observed. Thus, extensive effort is focused on developing strategies that cross the BBB and deliver active drugs to brain tumor cells. The use of nanocarrier systems, such as liposomes, micelles and nanoparticles (NPs), may be a better strategy for targeted drug delivery to glioblastoma cells [8–11].

As a new type of synthetic thin-shell structure, polymersomes self-assemble into vesicular structures based on amphiphilic block copolymers [12–14]. Compared with liposomes, polymersomes exhibit excellent stability and high membrane stability, and are promising alternatives to phospholipid-based vesicles [15–18]. Furthermore, due to the difference in acidity between solid tumors and surrounding normal tissues, pH-responsive polymersomes have been widely noticed and studied, and their great potential in more efficient delivery and rapid drug release in tumor cells has been described.

Low-density lipoprotein receptor-related protein 1 (LRP1) is expressed at high levels on the endothelial cells of brain capillaries and glioblastoma cells, but it is expressed at low levels in the normal brain parenchyma [19–21]. Based on these features, LRP1 might enable therapeutic agents to enter the central nervous system through the blood–brain barrier and become a potential therapeutic target for glioblastoma. Angiopep-2 (TFFYGGSRGKRNNFKTEEYC, ANG) is a peptide derived from the Kunitz domains of aprotinin that specifically binds LRP1 [22–24]. These characteristics make Angiopep-2 a promising candidate for LRP1-mediated targeted drug delivery to glioblastoma.

**Fig. 1:**
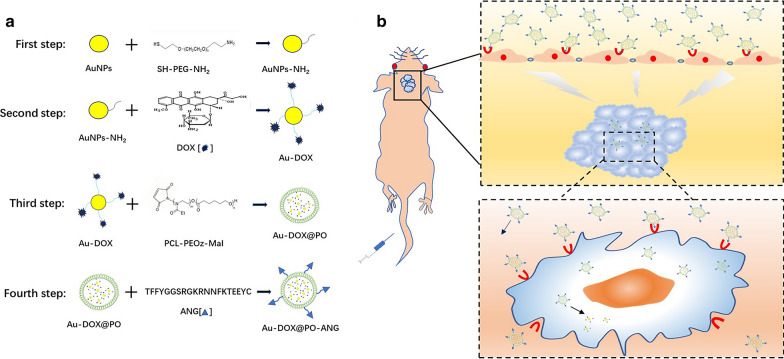
**a** Schematic representation of Au-DOX@PO-ANG synthesis. **b** Schematic Presentation of Angiopep-2-Conjugated pH-Sensitive Polymersomes (Au-DOX@PO-ANG) for Targeted Therapy for GBM

In this study, a new type of pH-sensitive polymersomes has been designed for glioblastoma therapy (Fig. 1a). First, the polymersomes are formed through the self-assembly of an amphiphilic coblock polymer, polycaprolactone-poly(2-ethyl-2-oxazoline) (PCL-PEOz). PEOz contains a tertiary amide group that readily binds to hydrogen ions in solution and forms hydrogen bonds with other tertiary amide groups in the PEOz molecule. Under acidic conditions, the formation of a large number of hydrogen bonds will disrupt the core–shell structure, thereby reducing the stability of the polymersomes and releasing the drug [25–27]. Second, gold nanoparticles and doxorubicin are designed as a complex drug. This type of complex drug has two characteristics. (1) Gold nanoparticles have been widely proven to be a radiosensitizer [28–30], which improve the radiotherapy (RT) effect of glioblastoma, and doxorubicin is an anthracycline antibiotic that is widely used to treat peripheral tumors [31]. Therefore, this complex drug has the function of radiotherapy and chemotherapy combined. (2) This type of complex drug ensures that gold nanoparticles and doxorubicin are encapsulated into the vesicles at the same time in certain proportions. More significantly, the encapsulation within polymersomes overcomes the problem of rapid clearance through the enhanced penetration and retention (EPR) effect of tumor cells [32–35]. The problem of a poor delivery capacity through the BBB is solved by modifying the Angiopep-2 peptide on the surface of the polymersomes. Angiopep-2-conjugated pH-sensitive polymersomes (Au-DOX@PO-ANG) enhance BBB transcytosis and glioblastoma-targeting ability (Fig. 1b), achieving a combination of radiotherapy and chemotherapy for U87-MG human glioblastoma xenografts in nude mice and significantly increased survival time.

## Results

### Characterization of the modified AuNPs.

AuNPs were synthesized by reducing the HAuCl_4_ solution with sodium citrate and modified with SH-PEG-NH_2_ to increase the stability and provide a group that will be coupled to DOX. TEM images of the unmodified AuNPs (Additional file 1: Figure S1a-1) and modified AuNPs (Additional file 1: Figure S1a-2) are shown in Additional file 1: Figure S1, and no obvious change in the morphology of SH-PEG-NH_2_ modified gold nanoparticles was observed. Based on the TEM images, the modified AuNPs showed good monodispersity and their size was approximately 10 nm. The hydrodynamic diameter of unmodified AuNPs was approximately 17.6 nm (Additional file 1: Figure S1b-1), and the diameter of modified AuNPs was approximately 22.4 nm (Additional file 1: Figure S1b-2). These results were similar to the TEM images when the effect of the hydrated outer shells was ignored.

The modified or unmodified AuNPs was characterized using UV–vis absorption spectroscopy and by measuring the ζ potentials to verify that the ligand was successfully modified. The ζ potentials and the absorption spectra of the modified and unmodified AuNPs are shown in Additional file 1: Figure S1c, d. The ζ potentials of modified AuNPs were − 24.6 ± 3.2 mV, and the results were significantly different from those of unmodified AuNPs, with a value of 27.5 ± 2.1 mV (Additional file 1: Figure S1c). UV–vis absorption spectroscopy documented the maximum absorption peak of unmodified AuNPs at approximately 520 nm, and significant changes were not observed after the modification (Additional file 1: Figure S1d). Based on these data, the AuNPs were successfully modified with SH-PEG-NH_2_ compounds.

The in vitro anti-GBM potency of modified AuNPs toward U87-MG GBM cells was evaluated using CCK-8 assays. As shown in Additional file 1: Figure S1e, more than 80% of cells remained viable after the addition of the modified AuNPs at concentrations up to 10 mg/L, which indicated that the modified AuNPs were nontoxic to GBM cells. The storage stability of the modified AuNPs in PBS (pH 7.4) at 4 °C is shown in Additional file 1: Figure S1f. The particle size exhibited little change over 4 weeks, which suggests a good storage ability.

### Verification of the conjugation of DOX on AuNPs

The modified AuNPs were coupled with DOX. First, the concentration and ratio of DOX should be determined to obtain suitable engineered polymersomes for the subsequent experiments. The IC50 of DOX, which inhibited the growth of U87-MG cells, was measured using the Cell Counting Kit-8 (CCK-8) (Additional file 1: Figure S2a). The IC50 was approximately 1.25 μg/mL at 48 h. Furthermore, the IC50 of AuNPs combined with radiotherapy at a dose of 6 Gy was approximately 4 nM (Additional file 1: Figure S2b). (Notably, 6 Gy is a common dose for radiotherapy according to previous reports [36]). Based on these results, the appropriate ratio of gold nanoparticles to DOX was determined as DOX: AuNPs = 1:80, which was used in the subsequent experiments.

TEM results did not reveal an obvious change in the morphology of gold nanoparticles after DOX coupling compared with nanoparticles that were not conjugated to DOX (Additional file 1: Figure S2c). The drug-loading rate of DOX was 92.89 ± 2.34%. The successful coupling was determined by recording UV–Vis absorption spectra and performing an in vitro drug release test. The absorption peak of DOX was observed in the Au-DOX spectrum (Additional file 1: Figure S2d). Moreover, the chemical composition of the Au-DOX was confirmed by Fourier transform infrared spectroscopy (FT-IR). The characteristic peaks of amide bond can be found in the spectra of Au-DOX (C = O: ν = 1690 cm^−1^ N–H: ν = 1590 cm^−1^ C-N: ν = 1190 cm^−1^) (Additional file 1: Figure S2e). The hydrodynamic diameter of the prepared Au-DOX was approximately 25.8 nm (Additional file 1: Figure S2f). The diameter was slightly larger than the modified AuNPs, but still less than 50 nm, which was conducive to encapsulation in polymersomes. These results indicated that the modified AuNPs were successfully coupled with DOX at pH 7.4 after preparation.

### Characterization of the Au-DOX@PO-ANG

**Fig. 2:**
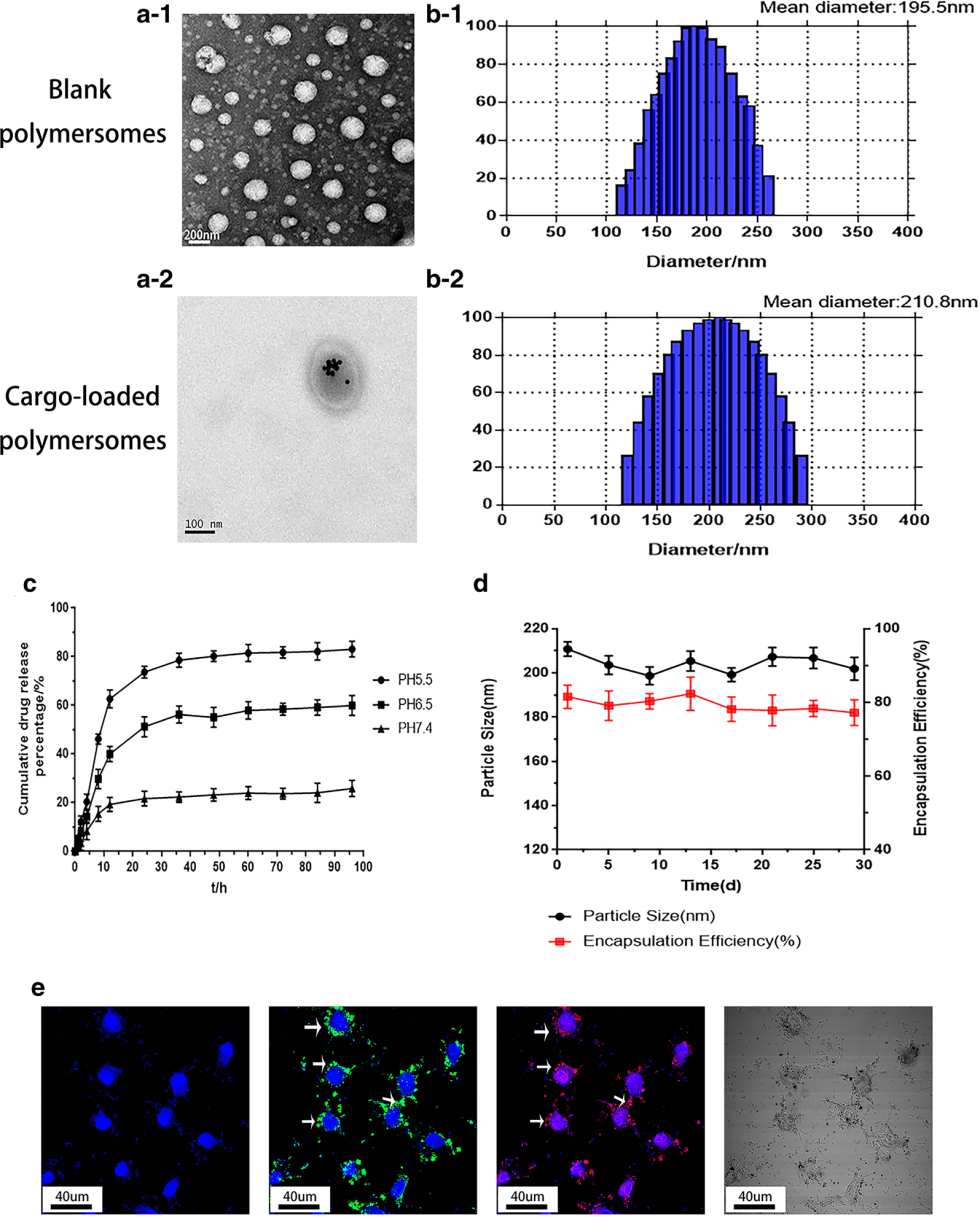
Characterization of the Au-DOX@PO-ANG. TEM images of blank polymersomes (**a-1**) and cargo-loading polymersomes (**a-2**). Size distribution and size in water of blank polymersomes (**b-1**) and cargo-loading polymersomes (**b-2**). **c** Cumulative release curve of Au-DOX from cargo-loading polymersomes under different pH conditions. **d** The storage stability of the cargo-loading polymersomes in PBS (pH 7.4) at 4 °C. **e** Au-DOX@PO-ANG directly measured by a super-resolution confocal microscope. The green fluorescence represents FITC carried on the ANG, the red fluorescence represents DOX and the blue fluorescence represents DAPI which is used to stain the nucleus of cells

Characterization of PCL-PEOz-maleimide is shown in Additional file 1: Figure S3. XPS spectra confirmed the existence of C–C, C = C, C-O, C = O, C-N bonds (Additional file 1: Figure S3a). ^1^HNMR spectrum determined the structure of the polymer. The characteristic peaks of PCL (δ = 1.40 ppm, δ = 1.65 ppm, δ = 4.04 ppm) and PEOz (δ = 1.12 ppm, δ = 2.29 ppm, δ = 3.46 ppm) are consistent with previous reports (Additional file 1: Figure S3b) [37, 38]. Photographs of blank polymersomes and cargo-loaded polymersomes are shown in Fig. 2a. The solution of the blank polymersomes was transparent, with a slight blue opalescence (Additional file 1: Figure S4a-1). After drug loading, the solution of cargo-loaded polymersomes was semitransparent and presented a light blue-violet color due to the encapsulation of the Au-DOX (Additional file 1: Figure S4a-2). TEM results revealed that the copolymer spontaneously assembled into polymersomes. The TEM images revealed a vesicle-like shape of blank polymersomes with average diameters of approximately 200 nm (Fig. 2a-1, Additional file 1: Figure S4b-1), consistent with the DLS results, and the Au-DOX complexes (black spot) were located inside the polymersomes, suggesting that Au-DOX complexes were encapsulated in the polymersomes (Fig. 2b-2, Additional file 1: Figure S4b-2). Additionally, the amount of Au-DOX complexes in the polymersomes was clearly regulated by the amount of Au-DOX complexes added. The mean hydrodynamic diameter of blank polymersomes was 195.5 nm (Fig. 2b-1). After Au-DOX loading and ANG coupling, the mean hydrodynamic diameter increased slightly to 210.8 nm (Fig. 2b-2).

The in vitro drug release of cargo-loaded polymersomes in PBS solutions was simulated at pH 7.4, 6.5 and 5.5 (Fig. 2c). In the release medium at pH 7.4, the release of DOX from the cargo-loaded polymersomes was stable, and the cumulative release rate was 25.89 ± 3.35% at 96 h, showing a certain sustained release effect. The release was accelerated at pH 6.5, and the cumulative release rate reached 59.98 ± 4.13% at 96 h. At pH 5.5, the cargo-loaded polymersomes showed a significant burst release, and the release was rapid and complete, with 83.11 ± 3.18% release at 96 h. The tumor tissue has a pH of approximately 6.5. The release of the drug was accelerated, which is beneficial to increase the drug concentration in the target cells or target tissues. CCK8 assays were performed to evaluate the anti-GBM potency of blank polymersomes and ANG-conjugated polymersomes toward U87-MG GBM cells in vitro. As shown in Additional file 1: Figure S4c, more than 80% of cells remained viable after treatment with blank polymersomes and ANG-conjugated polymersomes at concentrations up to 400 μg/mL, indicating that both blank polymersomes and ANG-conjugated polymersomes were nontoxic to GBM cells. The storage stability of the cargo-loading polymersomes in PBS (pH 7.4) at 4 °C is shown in Fig. 2d. The particle size and the encapsulation efficiency exhibited little change over 4 weeks, suggesting a good storage ability.

The prepared Au-DOX@PO-ANG suspension was also observed under a superresolution confocal microscope to confirm that ANG was conjugated on the surface of the polymersomes (Fig. 2e). The DOX encapsulated in the polymersomes appears red under a superresolution confocal microscope, enabling us to trace the position of the polymersomes. The FITC conjugated to ANG appears green under a superresolution confocal microscope. The prepared Au-DOX@PO-ANG suspension was observed under the laser-scanning confocal microscopy. The red and green fluorescence displayed extensive colocalization. The result indicated the presence of the ANG peptide on Au-DOX@PO-ANG.

### Quantification of the ANG peptide and Au-DOX in polymersomes

The coupling efficiency was calculated by dividing the amount of Angiopep-2 on the surface of polymersomes by the weight of the Angiopep-2 input. After the calculation, the standard concentration curve formula for the ANG peptide was y = 0.0044x-0.185 and the coupling efficiency was 11.3 ± 1.02%. The particle number of polymersomes was measured using NTA and yielded a value of 6 × 10^11^ particles/mL (Additional file 1: Figure S5). The number of ANG peptides connected to each polymersome was estimated to be 236. The drug loading capacity of Au-DOX in the polymersomes was approximately 5.3%, with an entrapment efficiency greater than 89.5% and an Au-DOX content of 0.725 mg/mL.

### Evaluation of the in vitro blood–brain barrier model

First, the purity of primary astrocytes (RA) and primary brain microvascular endothelial cells (BMECs) was analyzed using flow cytometry. As shown in Fig. 5a, the purity of primary astrocytes (RA) (Additional file 1: Figure S6a-1) and primary brain microvascular endothelial cells (BMECs) (Additional file 1: Figure S6a-2) reached more than 90% (94.8% and 90.2%). Second, we investigated whether LRP1 is overexpressed in the BBB endothelium and U87-MG GBM cells before evaluating the in vitro blood–brain barrier model. The levels of LRP1 in U87-MG cells, BMECs, and normal astrocytes were determined using Western blotting. The results corroborated that BMECs and U87-MG GBM cells overexpressed LRP1 (Additional file 1: Figure S6b, c). In comparison, LRP1 was not overexpressed on normal astrocytes (BMECs and normal astrocytes were prepared from newborn SD rats). Hence, the ANG peptide, which targets LRP1, is a potential ligand with which to achieve the high-efficiency targeting of GBM.

**Fig. 3:**
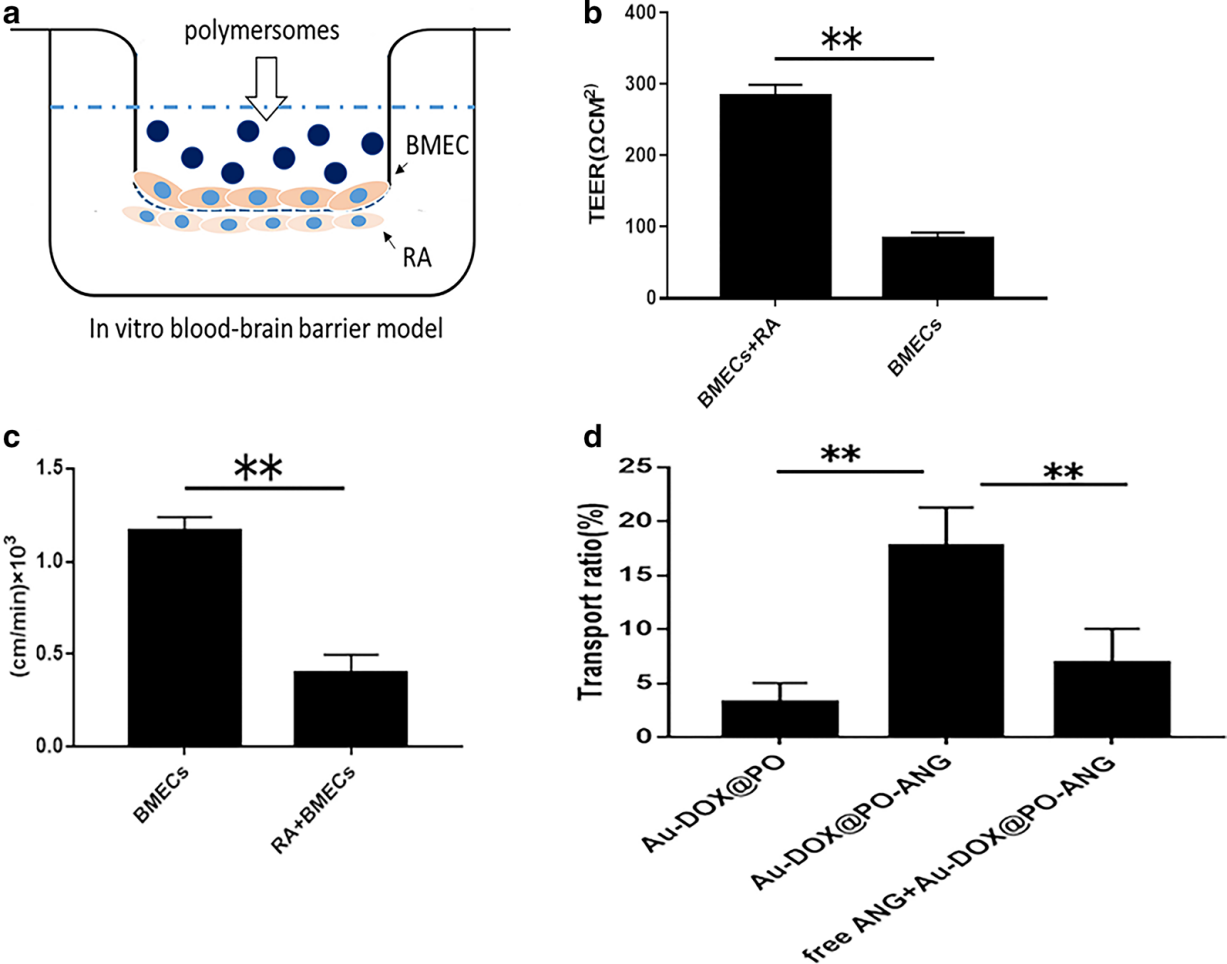
The targeting capability of Au-DOX@PO-ANG in vitro. **a** Illustration of polymersomes crossing the BBB model established using primary astrocytes and cerebral microvascular endothelial cells. **b** The TEER value of BBB models. **c** The permeability coefficient of sodium fluorescein in BBB models,**P < 0.01 compared with BMECs group. **d** The in vitro BBB model transport ratios (%) of Au-DOX@PO and Au-DOX@PO-ANG following 24 h of incubation, blockade experiments were conducted by pretreating U87-MG cells with free ANG (100 μg/mL, 30 min) before Au-DOX@PO-ANG incubation

Based on the aforementioned results, the in vitro blood–brain barrier model should be structured successfully. As shown in Fig. 3a, the in vitro blood–brain barrier model was constructed. Two experiments were used to evaluate the in vitro blood–brain barrier model. (1) Trans-endothelial electronic resistance was tested using a transmembrane resistance meter (Millicell ERS). (2) The permeability coefficient of the blood–brain barrier was determined. The TEER value of the in vitro blood–brain barrier model was 283.8 ± 15.79 (Ω × cm^2^), while the TEER value of the model built with BMECs alone was 82.7 ± 9.43 (Ω × cm2) (Fig. 3b). The permeability coefficient of the in vitro blood–brain barrier model to NaF was 0.396 ± 0.102 × 10^–3^ cm/min, which was lower than the permeability coefficient of the model built with BMECs alone (1.172 ± 0.069 × 10–3 cm/min) (Fig. 3c). These results indicate the in vitro blood–brain barrier model has been successfully established.

### Determination of the targeting capability of Au-DOX@PO-ANG in vitro

The ANG peptide plays two roles in this nanodrug delivery system: (1) it enables cargo-loaded polymersomes to penetrate the BBB and (2) it targets to glioblastoma in the brain.

The in vitro blood–brain barrier model was constructed successfully to evaluate the in vitro BBB transcytosis capability of Au-DOX@PO-ANG (Fig. 3a). The transport ratio of Au-DOX@PO-ANG was 17.74 ± 3.57% which showed a higher transport ratio compared with nontargeted Au-DOX@PO (3.24 ± 1.78%). Thus, the ANG peptide has a good BBB transport ability. As expected, the in vitro BBB transport ratio of Au-DOX@PO-ANG was substantially decreased by pretreating the in vitro BBB model with free ANG peptide (100 μg/mL), indicating that the BBB transcytosis of Au-DOX@PO-ANG was mainly mediated by LRP1 (Fig. 3d).

**Fig. 4:**
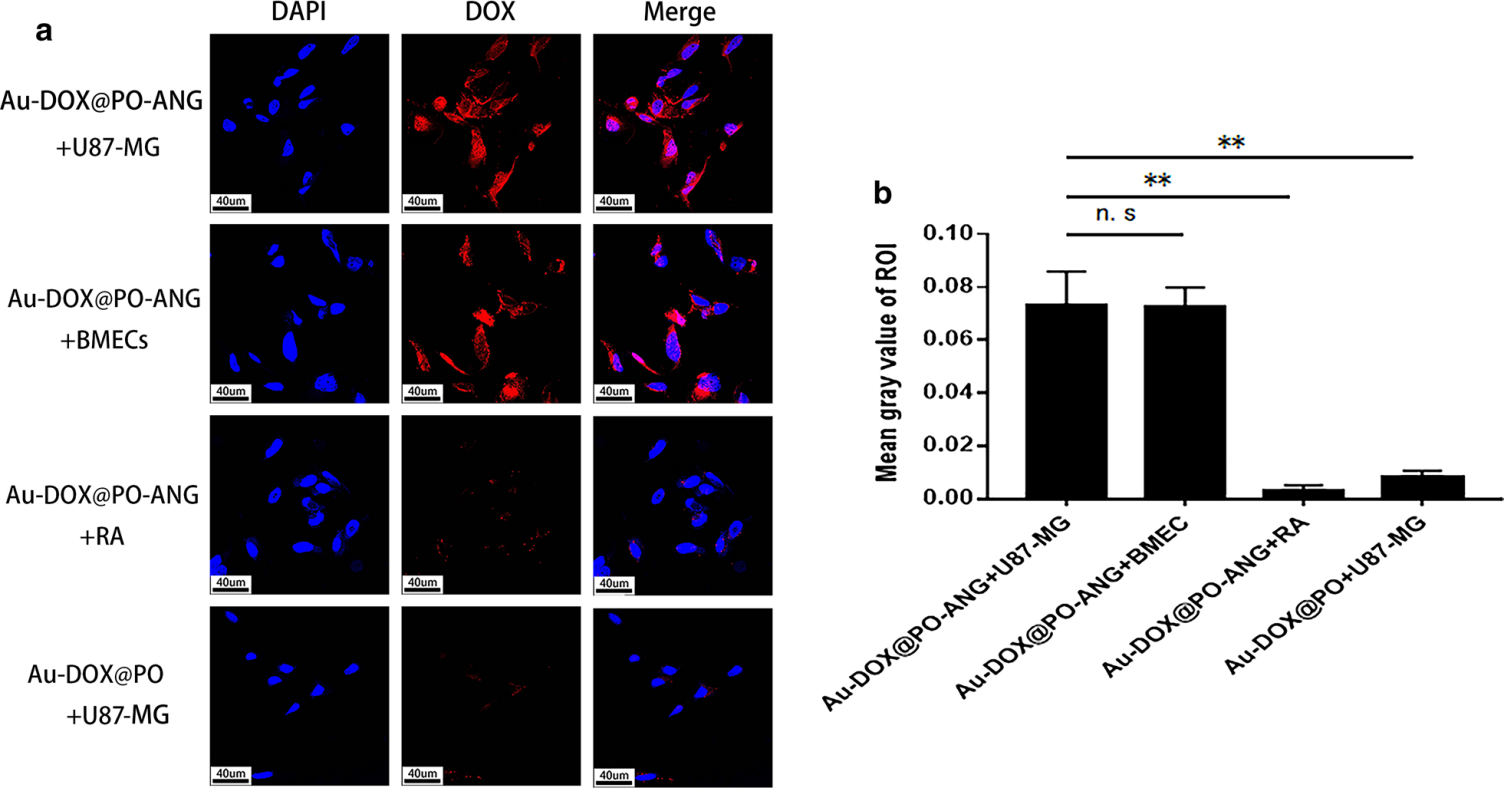
The targeting capability of Au-DOX@PO-ANG in vitro. **a** Target ability of Au-DOX@PO-ANG to glioblastoma in vitro. **b** Bar graph of ROI’s mean gray value, each bar represents the mean ± SD of six replicates, **P < 0.01

Next, the targeted or nontargeted polymersomes had been successfully prepared to investigate the LRP1-mediated endocytosis of Au-DOX@PO-ANG by U87-MG cells. The targeted or nontargeted polymersomes were incubated with U87-MG cells, BMECs (LRP1-positive cells) or astrocytes (LRP1-negative cells) for 4 h and then observed using a laser-scanning confocal microscopy. The DOX encapsulated in the polymersomes appears red. The following specific groups were established: (1) Au-DOX@PO-ANG + U87-MG cells, (2) Au-DOX@PO-ANG + BMECs, (3) Au-DOX@PO-ANG + RA, and (4) Au-DOX@PO + U-87 MG cells. As shown in Fig. 4, Au-DOX@PO-ANG-treated U87-MG cells exhibited strong cytoplasmic DOX fluorescence, supporting the fast cellular uptake and intracellular release of drugs. In contrast, nontargeted Au-DOX@PO-treated cells displayed noticeably less DOX fluorescence. Strong cytoplasmic DOX fluorescence was also observed in BMECs overexpressing LRP1, and less fluorescence was observed in astrocytes expressing LRP1 at low levels (Fig. 4a). Thus, we concluded that Au-DOX@PO-ANG specifically targeted U87-MG cells in vitro, and the targeting ability depended on the binding of the ANG peptide to LRP1. The semiquantitative analysis of fluorescence using ImageJ software (Fig. 4b) further confirmed the conclusions listed above. Moreover, a strikingly negligible amount of intracellular DOX was observed in the U87-MG cells treated with Au-DOX@PO, as no red signal was observed in the relevant imaging field. It was therefore considered that the nonspecific absorption of the nanoparticles could not be observed due to the short incubation time. When the incubation time was increased to 6 h, DOX fluorescence was observed in the nontargeted groups (Additional file 1: Figure S7).

### In vitro antitumor cytotoxicity of Au-DOX@PO-ANG

**Fig. 5:**
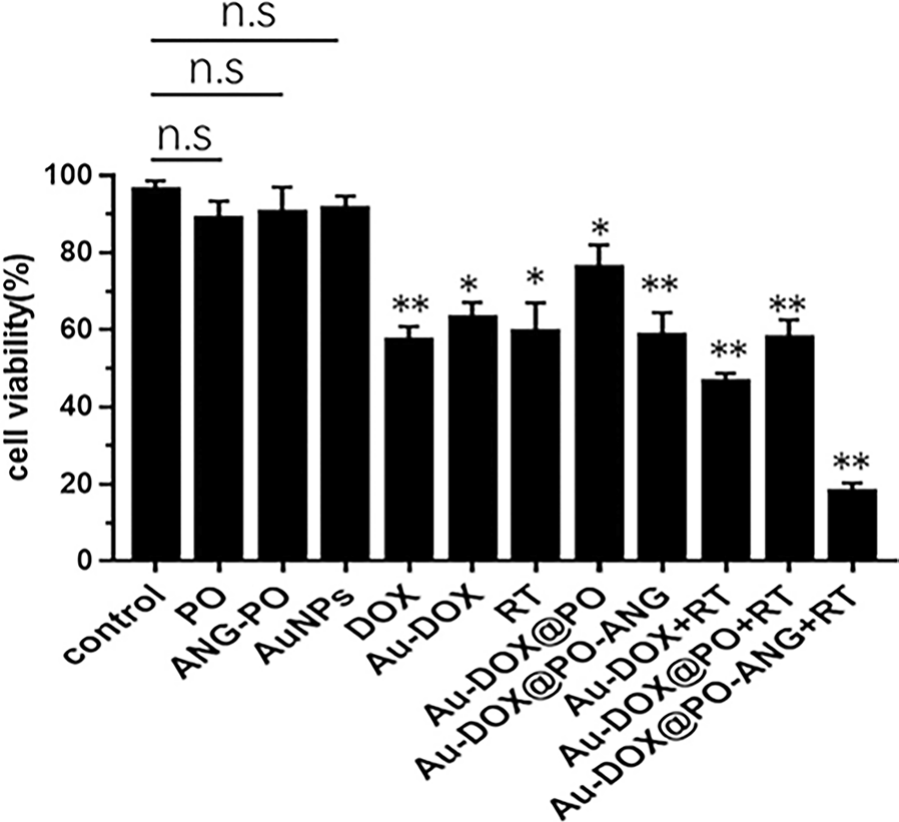
Treatment effect of Au-DOX@PO-ANG in vitro, each bar represents the mean ± SD of six replicates, *P < 0.05, **P < 0.01

The antitumor capability of Au-DOX@PO-ANG in U87-MG cells was evaluated using the Cell Counting Kit-8 (CCK-8). The in vitro antitumor activity of Au-DOX@PO-ANG was investigated to compare the treatment efficiency of free Au-DOX, Au-DOX@PO, and blank polymersomes, with and without radiotherapy. Cell viability was unchanged when blank polymersomes were applied alone or ANG-conjugated blank polymersomes were applied separately (Fig. 5). The administration of Au-DOX complexes or DOX alone resulted in a lower cell viability (63.2 ± 3.2% vs. 57.1 ± 3.1%), suggesting that the conjugation of DOX and AuNPs did not affect the antitumor activity of DOX. Only the incubation with Au-DOX@PO caused a slight reduction in cell viability (76.2 ± 4.8%). Thus, the toxicity of Au-DOX complexes was potentially limited by the encapsulation in polymersomes. However, the combination with ANG produced a more obvious anti-cancer activity, implying that ANG promoted drug uptake by tumor cells (58.7 ± 4.6%). The combined use of Au-DOX@PO-ANG and radiotherapy produced the most profound antitumor activity (18.1 ± 1.8% vs. Au-DOX@PO-ANG without radiotherapy: 58.7% ± 4.6%). Based on this finding, the inhibition of tumor cell growth was significantly increased through the combined effects of DOX and radiotherapy. The effect of the combination of DOX and radiotherapy was greater than the sum of the effects of the individual treatment.

### Determination of the targeting capability of Au-DOX@PO-ANG in vivo

**Fig. 6:**
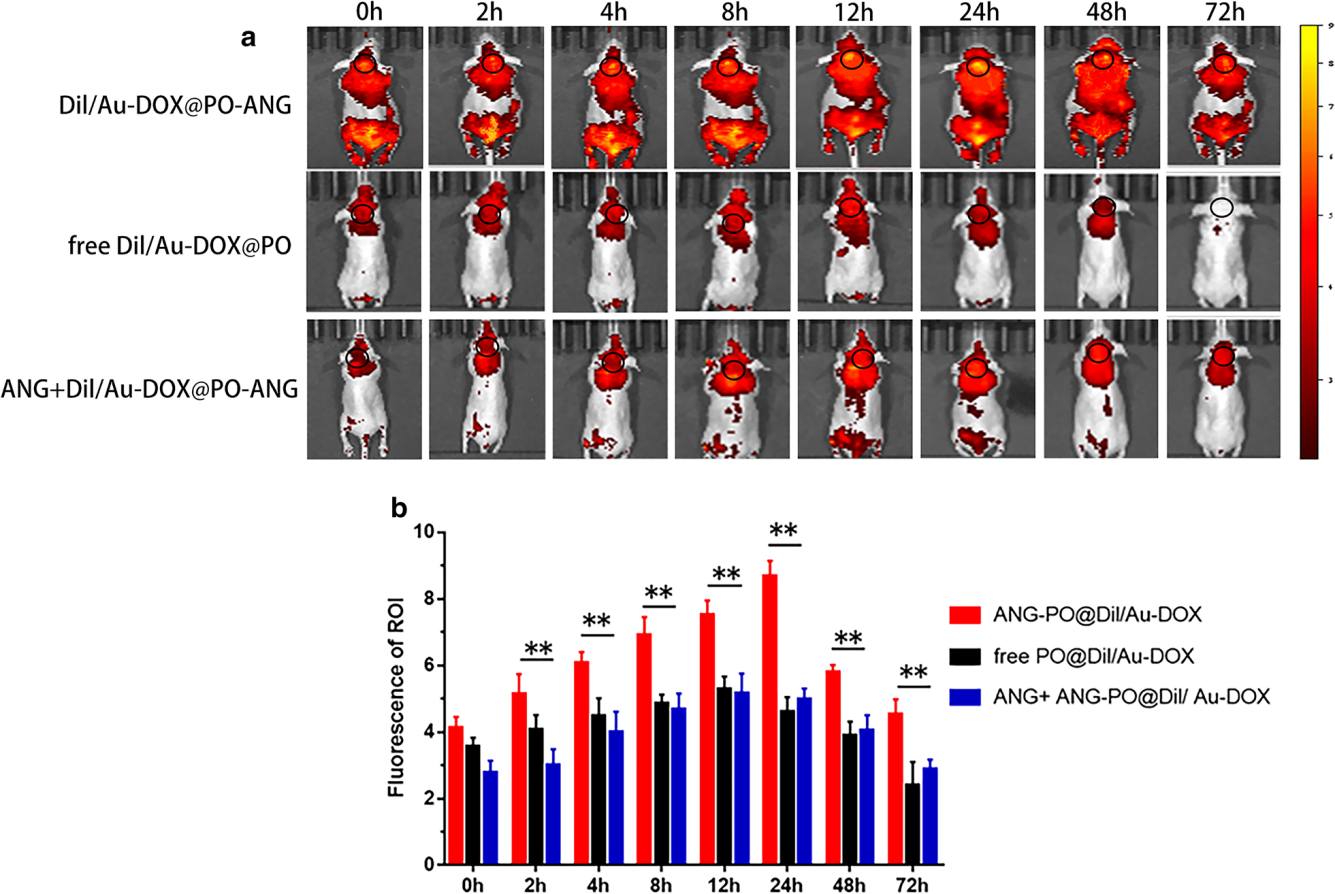
The targeting capability of Au-DOX@PO-ANG in vivo. **a** Distribution of Au-DOX@PO-ANG /free Au-DOX@PO in tumor-bearing mice. The smaller black circles are the tumor regions(ROI). **b** Bar graph of ROI's fluorescence, ** P < 0.01

NIR imaging was performed to detect the targeting ability of Au-DOX@PO-ANG in vivo. The near-infrared fluorescent dye DiI was encapsulated into the polymersomes to prepare DiI/Au-DOX@PO-ANG, along with the peptide blocking compound (ANG + DiI/Au-DOX@PO-ANG) and the free DiI/Au-DOX@PO, which was imaged as the control group. The method used to prepare these complexes was the same as Au-DOX@PO-ANG. The tumor-bearing nude mice were anesthetized with isoflurane before and during the experiment. Balb/c nude mice with orthotopically transplanted tumors were randomly divided into three groups: (1) DiI/Au-DOX@PO-ANG, (2) free DiI/Au-DOX@PO, and (3) ANG + DiI/Au-DOX@PO. NIR images were recorded at designated time points, including 0, 2, 4, 8, 12, 24, 48 and 72 h after the injection into the tail veins of the animals in the two groups (Fig. 6a); then, the fluorescence intensity of tumor regions (ROIs) was measured using ImageJ software (Fig. 6b). Very strong fluorescence was observed in the tumor area of the DiI/Au-DOX@PO-ANG group, while almost no obvious fluorescence was observed in tumor area of the free DiI/Au-DOX@PO group and the peptide blocking group. These results confirm the excellent ability of Au-DOX@PO-ANG to cross the blood–brain barrier and its good active targeting to the tumor area. The fluorescence of DiI/Au-DOX@PO-ANG in the tumor area peaked at 24 h. A small amount of fluorescence was observed up to 72 h, confirming its excellent persistence in the tumor area for a long time. These results show that this nano-drug delivery system has good targeting performance.

### Targeted therapy using Au-DOX@PO-ANG

**Fig. 7:**
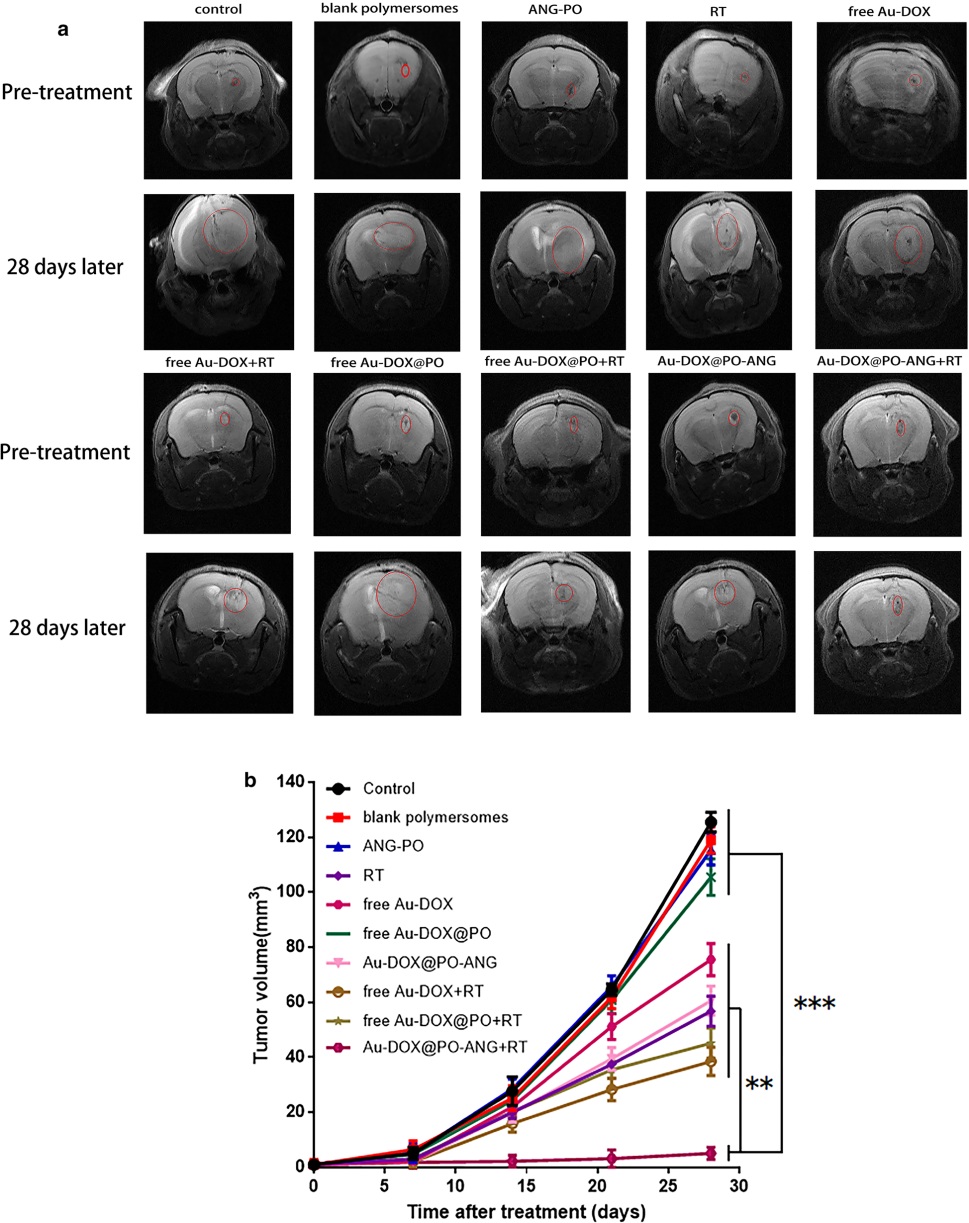
Treatment effect of Au-DOX@PO-ANG in vivo. **a** MIR images of each group before and 28 days after treatment, the regions in the red circles are the glioblastoma area. **b** The volume of orthotopic xenograft glioblastoma (6 mice each group) with different treatments. **P < 0.01, ***P < 0.001

The therapeutic effect of Au-DOX@PO-ANG on tumor-bearing mice was observed. Changes in tumor volume were observed using MRI. The rate of increase in the tumor volume slowed in the RT, free Au-DOX, Au-DOX@PO-ANG, free Au-DOX + RT, free Au-DOX@PO + RT and Au-DOX@PO-ANG + RT groups (P < 0.05). A more remarkable effect was observed in the Au-DOX@PO-ANG + RT group (P < 0.001), and a significant increase in tumor volume was not observed when comparing the images captured treatment and after 28 days of treatment (Fig. 7a).

**Fig. 8:**
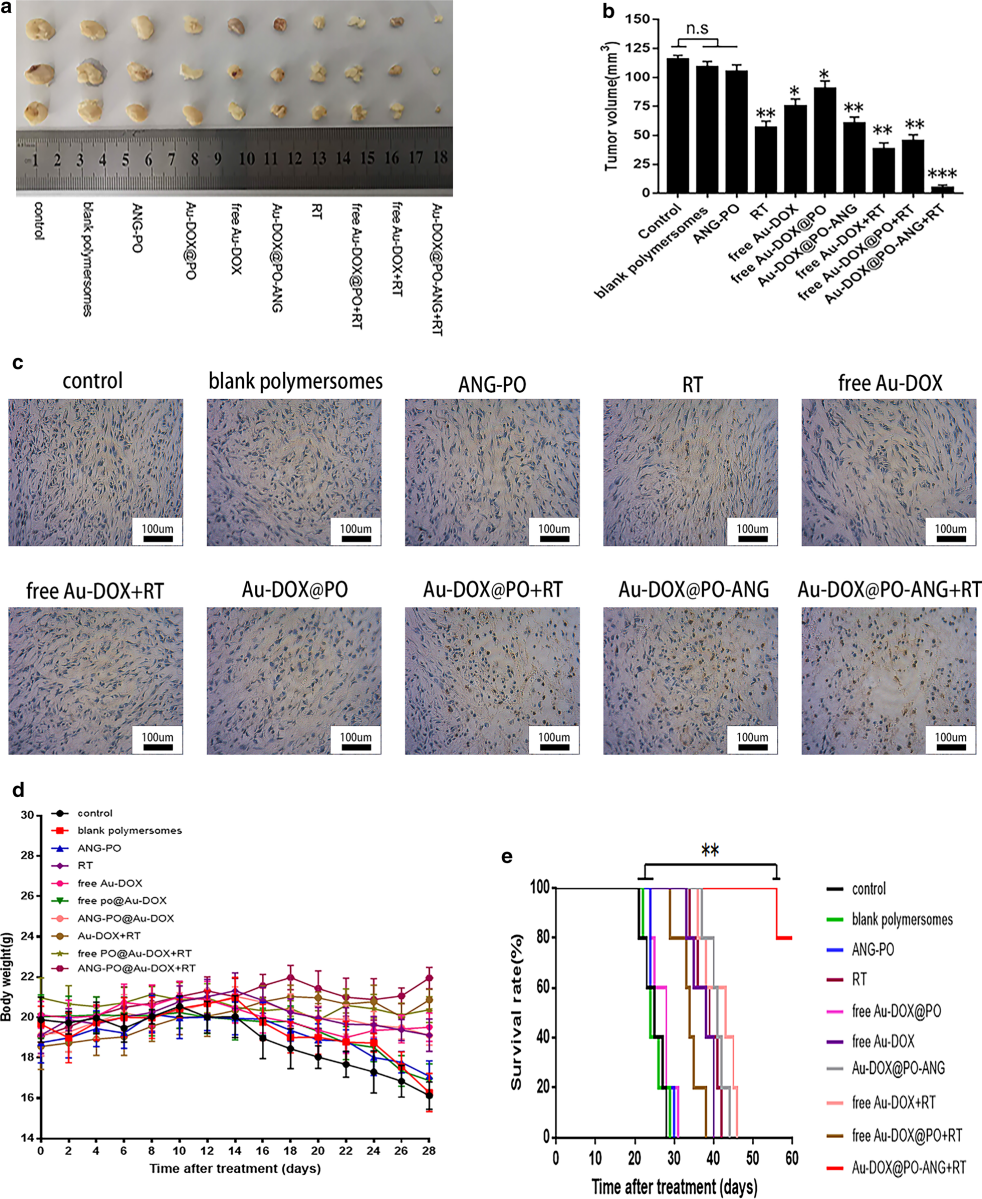
**a** The gross morphology and diameter of tumors ex vivo. **b** The volume of tumors ex vivo. **c** TUNEL assays of U87-MG glioblastoma sections from rats receiving different therapies after the treatments. Brown staining indicated apoptotic cells. **d** The weight changes of tumor-bearing nude mice in each group during treatment. **e** KaplaneMeier survival curve of U87 tumor-bearing mice, n = 6 mice per group. Data are the mean ± SD, **P < 0.01

The tumor growth rate was slightly slower in the RT and free Au-DOX@PO + RT groups compared with the control group, potentially due to the effect of radiotherapy, while the changes observed in the free Au-DOX and Au-DOX@PO-ANG groups may be due to a small amount of DOX entering the tumor area through the blood–brain barrier. The free Au-DOX + RT group possesses both radiotherapy and chemotherapy activity, but the effects are not ideal because the majority of nanomedicines are blocked by the blood–brain barrier. In contrast, the tumor sizes in animals treated with blank polymersomes, ANG-PO and free Au-DOX@PO increased significantly compared to the pretreatment value (P < 0.05). These results suggest that the therapeutic effect is limited without drug carriers and targeted modifications. (Figs. 7b, 8a, b).

Immunohistochemical TUNEL staining was performed using paraffin-embedded tumor tissue sections (Fig. 8c). TUNEL assays were conducted to correlate cell apoptosis with increased levels of cleaved caspase-3 in tumor tissues. Apparently, the most significant increase in cell apoptosis was observed in the Au-DOX@PO-ANG + RT group among all groups, and this result is consistent with the MRI data.

Furthermore, we also observed changes in the weights of tumor-bearing nude mice in each group during treatment. The weight of tumor-bearing nude mice decreased significantly in the blank polymersomes, ANG-PO and free Au-DOX@PO groups after 28 days of treatment, indicating the existence of significant cachexia. No significant changes were observed in the body weights of animals in the Au-DOX@PO-ANG + RT group (Fig. 8d).

### Evaluation of the survival period

Overall survival was recorded to evaluate the long-term effect of Au-DOX@PO-ANG + RT. The Kaplan–Meier survival curves were drawn according to the average survival time and standard deviation (Fig. 8e). The Au-DOX@PO-ANG + RT group survived for a much longer period than the other groups. The single radiotherapy groups (RT and free Au-DOX@PO + RT) exhibited longer survival than the control group due to the effect of radiotherapy, while the single chemotherapy groups (free Au-DOX and Au-DOX@PO-ANG) exhibited longer survival than the control group due to the small amount of DOX entering the tumor area. Furthermore, the survival period of the free Au-DOX + RT group was extended due to the effects of both radiotherapy and chemotherapy, but the effect was limited because the majority of nanomedicines was blocked by the blood–brain barrier. According to the above results, improving tumor targeting and combined application of radiotherapy and chemotherapy are effective methods to improve the curative effect.

### Evaluation of the safety of Au-DOX@PO-ANG in vivo

**Fig. 9:**
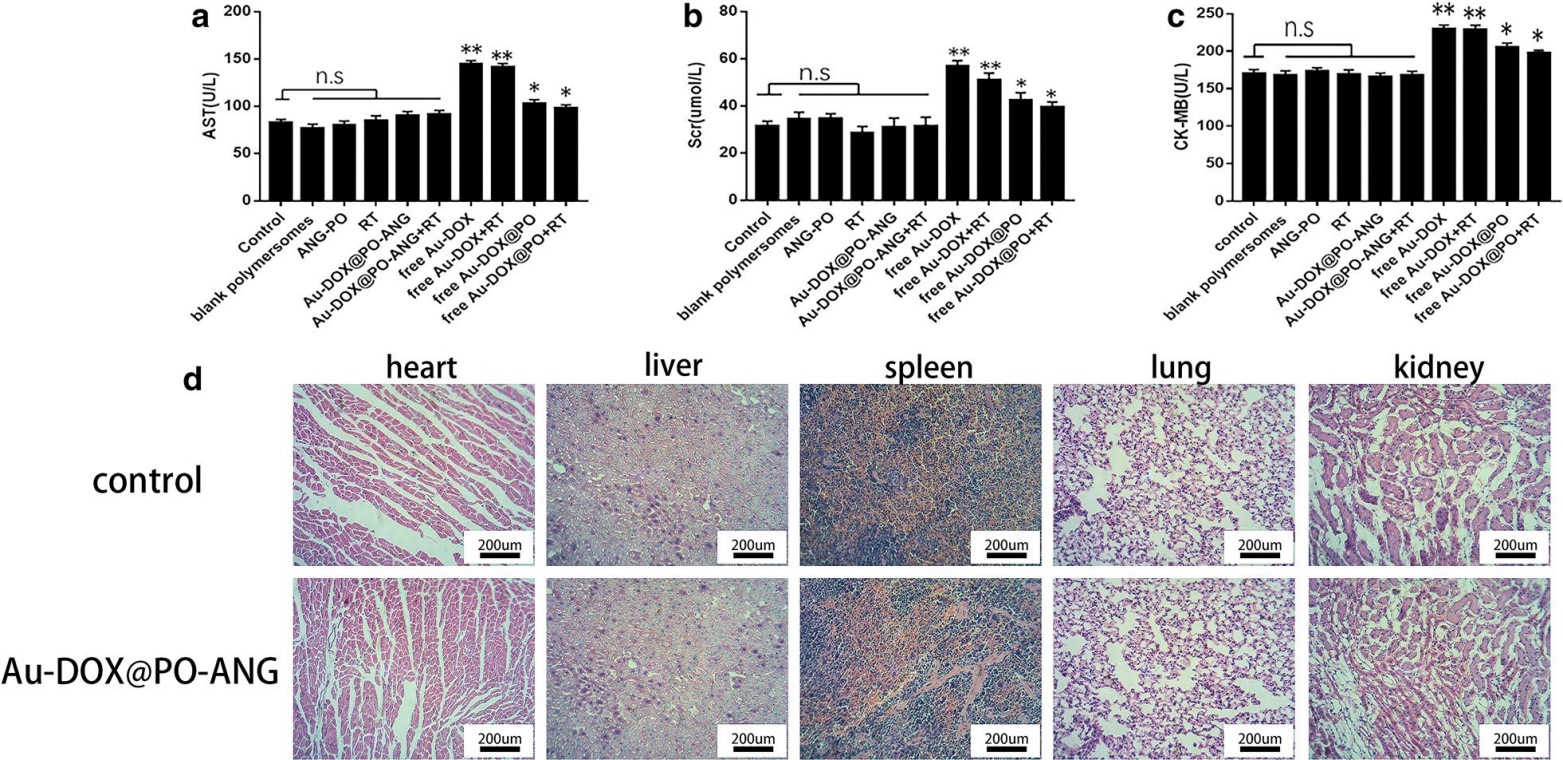
Safety evaluation of Au-DOX@PO-ANG in vivo. Blood biochemical indicators of each treatment group, and the activities of (**a**) AST, (**b**) Scr, and (**c**) CK-MB were measured. **d** HE staining analysis of the main organs

The levels of blood biochemical indicators in each treatment group revealed obvious side effects of Au-DOX, as higher levels of CK-MB, AST and Scr were observed than in the PBS group (P < 0.05). The toxicity and side effects of Au-DOX were significantly reduced after encapsulation in polymersomes. Targeted polymersomes further reduced the toxic and side effects of Au-dox, similar to the “nontoxic” level of the PBS group (Fig. 9a–c). Furthermore, the results of HE staining did not reveal significant pathological changes in the main organs of the Au-DOX@PO-ANG group, such as heart, liver, spleen, lung and kidney, compared with the PBS group (Fig. 9d). These results confirmed the safety of Au-DOX@PO-ANG in vivo.

## Discussion

In the clinic, the main treatment strategies for glioblastoma are mainly surgery supplemented by radiotherapy and chemotherapy [4]. However, the therapeutic effects are usually limited. The reasons for the limited effects can be summarized as follows: (1) because of the high invasiveness of tumor cells, the tumor cannot be completely removed by surgery; (2) the existence of the blood–brain barrier prevents chemotherapy drugs from entering the brain, and thus, the low concentration of chemotherapy drugs in the tumor area leads to unsatisfactory therapeutic effects; and (3) relevant studies have shown that glioblastoma has low sensitivity to radiotherapy, which limits its effects [28].

In this study, we focused on solving the second and third points listed above. To be more specific, our main goal consist of two parts: (1) overcoming the blood–brain barrier and increasing chemotherapy drug concentrations in the tumor area and (2) enhancing the sensitivity of glioblastoma cells to radiotherapy.

Various nanotechnology-based approaches, such as the use of micelles, liposomes, dendrimers and solid lipid nanoparticles have been studied for glioblastoma treatment [39]. Liposomes have been extensively tested for drug delivery, and various formulations have been examined in clinical trials [40]. Polymeric carriers (polymersomes) are superior to liposomes in terms of their better loading capability, longer blood circulation time, reduced drug leakage and larger storage capacity [41]. Based on the above theoretical basis, we designed a targeted nanodrug delivery system mediated by LRP1 (Au-DOX@PO-ANG). The polymersomes we used in this study show pH sensitivity compared with traditional drug carriers, and can also be loaded with nanomaterials and chemical drugs. However, polymersomes lack reliable targeting abilities and cannot achieve targeted delivery to brain tumors. We modified the surface of polymersomes with the polypeptide Angiopep-2 by chemical bond coupling to achieve targeted delivery of the polymersomes to glioblastoma. Studies on ANG-functionalized drug delivery systems have demonstrated that ANG has a higher BBB transcytosis efficacy and glioblastoma accumulation than lactoferrin, transferrin, and avidin [42–45]. In addition to these, gold nanoparticles and doxorubicin were designed as complex drugs that combine radiotherapy and chemotherapy. This type of complex drug ensured that the gold nanoparticles and doxorubicin could be encapsulated into the vesicles at the same time in certain proportions.

In this study, first, the size, shape and stability of AuNPs, Au-DOX, Au-DOX@PO and Au-DOX@PO-ANG were characterized and measured respectively. Additionally, the quantity of the ANG peptide and Au-DOX in Au-DOX@PO-ANG were measured. The results showed that this type of nano-drug delivery system had good stability, which suggested its potential application in vivo. Furthermore, the in vitro drug release from the cargo-loaded polymersomes suggested that this type of nanodrug delivery system has pH-sensitivity, which is beneficial for increasing the drug concentration in the target cells or target tissues.

Next, the targeting ability of Au-DOX@PO-ANG at the cellular level was further evaluated. The ANG peptide plays two roles in this nanodrug delivery system: (1) it enables cargo-loaded polymersomes to penetrate the BBB. To validate this effect, we constructed in vitro blood–brain barrier model to evaluate its in vitro BBB transcytosis capability. The result has shown that ANG peptide has a good BBB transport ability. (2) it targets glioblastoma in the brain [46]. To prove its target effect, targeted or nontargeted polymersomes were successfully prepared and used in laser confocal imaging experiments to investigate the LRP1-mediated endocytosis of Au-DOX@PO-ANG by U87-MG cells. By detecting the fluorescence intensity during this experiment, we verified the glioblastoma’s targeting ability. In addition to in vitro experiments, the result of in vivo near infrared imaging experiments showed that, compared with non-targeting and the peptide blocking group, a much stronger fluorescence was observed in the tumor area of the targeting group. These results confirmed the excellent abilities of Au-DOX@PO-ANG to cross the blood–brain barrier and to actively target the tumor area.

Encouraged by these results, targeted therapy experiments were conducted in vitro and in vivo. For in vitro ones, the antitumor capability of Au-DOX@PO-ANG in U87-MG cells was evaluated using the Cell Counting Kit-8 (CCK-8) assay. Based on the results, the inhibition of tumor cell growth was significantly increased through the combined effects of DOX and radiotherapy, which suggested that the effects of the combination treatment was greater than the sum of the effects of the individual treatments. For in vivo ones, the therapeutic effect of Au-DOX@PO-ANG on tumor-bearing mice was also observed. Excitingly, there was a significant synergetic effect from radiotherapy combined with DOX, which greatly improved the curative efficacy.

In addition to these ideal findings, we also found some unexpected results. For example, in a targeted therapy in vivo experiment, the therapeutic effects of free Au-DOX were better than those of Au-DOX@PO, potentially because a small amount of Au-DOX entered the brain through the intercellular space and exerted a therapeutic effect due to its small particle size [47].

In order to deepen our studies, the long-term effects of Au-DOX@PO-ANG used in vivo need continuous observation, and the optimal dose, treatment time and monitoring of RT are all worthy of further research. After optimization, the effects of Au-DOX@PO-ANG will be maximally achieved.

## Conclusions

In summary, a new targeted drug delivery system was fabricated for the treatment of glioblastoma. In this delivery system, gold nanoparticles, which are an ideal radiosensitizer, and the chemotherapy drug doxorubicin were coupled to form a compound drug, ensuring that each polymersome was loaded with the same proportion of gold nanoparticles and doxorubicin. The results of in vitro drug release studies showed that this delivery system had pH-sensitivity and the ability to respond to the tumor microenvironment. Furthermore, these targeted polymersomes crossed the blood–brain barrier both in vitro and in vivo. Based on the results of the in vivo experiments, these cargo-loaded polymersomes conjugated with the Angiopep-2 peptide were capable of targeted combination chemotherapy and RT and exhibited a noticeably better antitumor efficacy than chemotherapy or radiotherapy alone, without any noticeable systemic toxicity. Moreover, the gold nanoparticles further increased the therapeutic effect of radiotherapy. Thus, these targeted cargo-loading polymersomes potentially represent a promising nanoplatform for future translational research in glioblastoma therapy.

## Experimental section

### Materials

U87-MG cells were purchased from Shanghai Cell Bank of Chinese Academy of Sciences (Shanghai, China). All media and reagents for cell culture were purchased from Gibco (Carlsbad, CA, USA). PCL-PEOz-maleimide and SH-PEG-NH_2_ were purchased from Xi'an Ruixi Biological Technology Co., Ltd. DOX was purchased from Sigma-Aldrich. The ANG peptide (conjugated with FITC) was purchased from KareBay Biochem, Inc. (Ningbo, China).

### Preparation and characterization of the modified AuNPs and Au-DOX

AuNPs were prepared using a previously reported method, with a slight modification [48]. Briefly, a mixture containing 1 mL of HAuCl_4_ (1%, w/w) and 100 mL of ultrapure water was brought to reflux with stirring, and then 4 mL of a sodium citrate trihydrate solution (1%, w/w) were added quickly, changing the color of the solution from yellow to deep red. After the color became wine red, the solution was heated for an additional 15 min, cooled to room temperature and finally placed in a refrigerator for storage. After preparation, gold nanoparticles were modified with SH-PEG-NH_2_ to increase their stability. The specific procedure was to add 1 mL of an SH-PEG-NH_2_ solution with a concentration of 5 × 10^–4^ mol/L to the prepared gold nanoparticles solution, and then thoroughly mix the two at room temperature. After the reaction, the solution was centrifuged at 12,000 rpm, 25 ℃ or 10 min, the supernatant was removed, and the precipitate was mixed and then stored at 4 °C. Specifically, the AuNPs and doxorubicin solution were conjugated via natural reactions between the amino group and carbonyl group in a 37 ℃ bath for a 4-h incubation period, and then the solution was centrifuged at 12,000 rpm for 10 min. The supernatant was removed and the precipitate was mixed. All glassware used throughout the process was soaked in aqua regia (3 parts HCl and 1 part HNO_3_) for 24 h, rinsed with water and then oven dried before use. All solutions were prepared with ultrapure water. Transmission electron microscopy (TEM) images were captured with an H-7650 transmission electron microscope (Tokyo, Japan). UV–vis absorption spectra of the AuNPs were obtained using an ultraviolet–visible spectrophotometer (UV–Vis spectrophotometer, UV-3600, Shimadzu, Tokyo, Japan) at an absorbance of 540 nm. The hydrodynamic diameter of the AuNPs was measured using dynamic light scattering (DLS) (Brookhaven Instruments Co., Holtsville, NY, USA). Detailed descriptions of the experimental setup are provided in another study [49]. The biosafety of gold nanoparticles was evaluated using the CCK8 assay.

### Preparation and characterization of cargo-loaded pH-responsive polymersomes

The pH-responsive polymersomes were prepared with a blend of mPCL-PEOz-maleimide using the thin-film rehydration method. Briefly, 10 mg of mPCL-PEOz-maleimide were dissolved in 4 mL of dichloromethane (CH_2_CL_2_). The solution was evaporated at room temperature to form a dry lipid film. Then, the round-bottomed flask was cooled to a 40 °C in a vacuum oven for 8 h to remove the residual organic solvent. Then, the film was hydrated with ultrapure water mixed with Au-DOX at 60 °C for 6 h to obtain Au-DOX@PO. Blank polymersomes were prepared using a similar method. The morphology of blank polymersomes and cargo-loaded pH-responsive polymersomes was observed using an H-7650 transmission electron microscope (Tokyo, Japan) after negative staining with a 1% uranyl acetate solution. The average diameters of blank polymersomes and cargo-loaded pH-responsive polymersomes were determined by performing dynamic light scattering measurements using a Nano ZS Zetasizer (Malvern Instruments, Malvern, UK).

### Conjugation of Angiopep-2 to cargo-loaded pH-responsive polymersomes

Cargo-loaded pH-responsive polymersomes were incubated with 50 μg of Angiopep-2 and stirred at a low speed for 12-h under a pure nitrogen atmosphere at room temperature. During this process, maleimide groups on the surface of the polymersomes specifically reacted with the thiol groups of Angiopep-2. After the reaction was complete, the unbounded polypeptide was removed by ultrafiltration with ultrafiltration tube at room temperature, followed by centrifugation at 4000*g* for 30 min.

### Verification and quantification of the amount of Angiopep-2 conjugated on the surface of polymersomes

Several experiments, which are described below, were conducted to verify whether Angiopep-2 was successfully conjugated on the surface of polymersomes. The targeted cargo-loaded complexes were incubated with U87-MG cells, and the distributions of the two types of fluorescence signals (DOX contained in the complexes and FITC conjugated to Angiopep-2) were assessed with a confocal microscope. Angiopep-2 was quantified using a reported method [50]. After ultrafiltration, the supernatant was collected and the absorbance was measured at 200 nm using a ultraviolet–visible spectrophotometer. The coupling efficiency was obtained by dividing the amount of Angiopep-2 on the surface of polymersomes by the weight of the Angiopep-2 input. The surface density of Angiopep-2 was determined by dividing the number of Angiopep-2 molecules by the calculated average number of polymersomes using the methods described by Olivier et al. [51].

### In vitro pH-responsive release of ANG-PO@Au-DOX

Two milliliters of the Au-DOX@PO-ANG solution were placed in a dialysis bag (MWCO 1500), which placed in 50 mL of phosphate buffer at different pH values (pH 7.4, pH 6.5, and pH 5.5). Then, 2 mL of the dialysate sample was removed at a specific time points (0.5, 1, 2, 4, 8, 12, 24, 36, 48, 60, 72, 84, or 96 h) and immediately returned to maintain the volume of the medium. The absorbance of DOX in the dialysate was measured at 480 nm using a UV–Vis spectrophotometer. The in vitro release profile was plotted.

### Storage stability of Au-DOX@PO-ANG

An experiment was performed to verify the storage stability of Au-DOX@PO-ANG, as described below. The measurement was performed at 4 °C for 4 weeks. The particle size and drug encapsulation efficiency were detected at 0 h, 8 h, 16 h, 24 h, 1 day, 7 days, 14 days, and 21 days. The storage stability profile was plotted.

### Cell culture and reagents

In the present study, the human glioblastoma multiforme cell line U87-MG purchased from the Shanghai Cell Bank of Chinese Academy of Sciences (Shanghai, China) was cultured in Dulbecco’s Modified Eagle’s Medium (DMEM) containing 10% FBS and antibiotics.

### Establishment of an in vitro blood–brain barrier model

The in vitro blood–brain barrier model was established using a previously reported method [52]. Briefly, glial cells and cerebral vascular endothelial cells were prepared from newborn SD rats. The transwell chamber was flipped, the density of astrocytes was adjusted to 1 × 10^5^ cells/mL, and cells were inoculated on the back of the transwell chamber in a 6-well plate. After 4 h of culture, the chamber was flipped and then the solution was replaced once every 2 days. After the astrocytes reached 80% confluence, endothelial cells were inoculated on the front side of the chamber. The specific steps were to adjust the cell density to 5 × 10^4^ cells/mL and inoculate the cells on the front surface of the gelatin-coated 6-well plate. The medium was changed for 2 days, and the solution was changed once every 2 days.

### Verification that the complexes crossed the blood–brain barrier in vitro

Au-DOX@PO and Au-DOX@PO-ANG were added to the donor chamber (polymersome concentration: 0.2 mg/mL). A competition experiment was conducted by pretreating the in vitro blood–brain barrier model with free ANG (100 μg/mL) for 0.5 h before adding Au-DOX@PO-ANG. The liquid in the lower chamber was collected at the time points of 6, 12 and 24 h. Eight hundred microliter aliquots were removed from the basolateral compartment and replaced with an equal volume of fresh medium, and the collected samples were immediately stored at 4 ℃. The diluted samples were collected sequentially according to the time of collection. The transport ratio (%) of polymersomes was calculated as the amount of Au-DOX@PO-ANG that accumulated in the basolateral compartment after crossing the monolayer to the initial amount.

### Antitumor effects of Au-DOX@PO-ANG in vitro

After considering the two types of therapeutic effects (RT and DOX) and the coupling relationship between AuNPs and doxorubicin, we conducted this experiment using two groups to evaluate the targeting effect of each treatment and whether RT and DOX exerted a synergistic effect when administered in combination: the Au-DOX group and Au-DOX + RT group. The detailed groups are listed follows: (1) control, (2) blank polymersomes, (3) ANG-PO, (4) AuNPs, (5) DOX, (6) free Au-DOX, (7) RT, (8) free Au-DOX@PO, (9) Au-DOX@PO-ANG, (10) free Au-DOX + RT, (11) free Au-DOX@PO + RT, and (12) Au-DOX@PO-ANG + RT. The concentration of Au-DOX was 100 μg/mL in each group (the concentration ratio of AuNPs and doxorubicin was 80:1). In every treatment group, cells were incubated with the “treatment” for 24 h. Subsequently, cells were treated with radiotherapy at a dose of 6 Gy, and then incubated in the cell culture incubator for 24 h. Afterwards, the optical density (OD) value of the cells was measured using the Cell Counting Kit-8 (CCK-8). The percent inhibition of cell growth in each treatment group was calculated as follows: percent inhibition = (OD of the control group−OD of the experimental group)/OD of the control group × 100%, where the control group was the PBS-treated group.

### Establishment of an orthotopic glioblastoma-bearing nude mouse model

Five-week-old female BALB/c nude mice were purchased from Nanjing Institute of Biomedical Research. All the operations were performed under 1% pentobarbital anesthesia, and every effort was made to reduce the suffering of the animals. The human glioblastoma cell line (U87-MG, 1 × 10^5^ cells in 5 µL of PBS) was transplanted into the right striatum to construct an orthotopically transplanted tumor model. The specific steps are listed below. The nude mice were anesthetized and fixed in the stereotactic apparatus for small animals. The scalp was incised at the middle of the head and separated, the periosteum was removed, and the anterior fontanelle was exposed as the coordinate origin. Next, the insertion point was 1.8 mm to the right and 1.0 mm to the front of the anterior fontanelle. In the final step, 5 µL of the cell suspension (1 × 10^5^ cells) was injected into the striatum at a rate of 1 µL/minute, the needle was maintained in place for 3 min, and the incision was sterilized and sutured after the syringe was slowly removed.

### Treatment groups and protocol for the administration of Au-DOX@PO-ANG in vivo

The antitumor efficacy of the nano-system combined with radiotherapy was evaluated in the orthotopic glioblastoma-bearing nude mouse model. The mice were randomly divided into ten groups (n = 6): (1) control, (2) blank polymersomes, (3) ANG-PO, (4) RT, (5) free Au-DOX, (6) free Au-DOX@PO, (7) Au-DOX@PO-ANG, (8) free Au-DOX + RT, (9) free Au-DOX@PO + RT, and (10) Au-DOX@PO-ANG + RT. The dose of Au-DOX was 800 µg/200 µL PBS. Each reagent was injected via the tail vein. Twenty-four hours later, the radiotherapy groups were exposed to radiation at 6 Gy. The treatment was administered a total of 7 times. Meanwhile, MRI was performed every other day from the beginning of treatment to 28 days after treatment, and the tumor length (L) and width (W) were measured. The tumor volume (V) was calculated using the formula: V = π/6 × L × W^2^. The curative effect was evaluated with this parameter, and the control group was the PBS-treated group.

## Statistical analysis

The data are presented as the means ± standard deviations (SD) and were analyzed using GraphPad Prism version 7.0 software (GraphPad Software, Inc., San Diego, CA, USA). The data from two groups were compared using Student’s t-test. The survival analysis was performed by constructing Kaplan–Meier curves. The differences between the two groups were considered statistically significant at *P < 0.05, and very significant at **P < 0.01 and ***P < 0.001.

## Supplementary Information

**Additional file 1: Fig. S1.** Characterization of AuNPs, AuNPs-NH2. TEM images of AuNPs (a-1) and AuNPs-NH2 (a-2). Size distribution and size in water of AuNPs (b-1) and AuNPs-NH2 (b-2). c Zeta-potentials of AuNPs and AuNPs-NH2. d Spectrophotometer results of AuNPs and AuNPs-NH2. e In vitro toxicity evaluation of U87-MG cells after co-incubation with AuNPs-NH2 for 24 h by Cell Counting Kit-8 (CCK-8) method. f Dynamic light scattering (DLS) size measurements of AuNPs-NH2 in PBS for varied time durations (0 ~ 29 days).** Fig. S2.** Characterization of Au-DOX. IC50 of DOX (a) and AuNPs combined with radiotherapy of 6Gy (b). c TEM image of Au-DOX. d Spectrophotometer results of AuNPs , AuNPs-NH2 and Au-DOX. e FT-IR spectrum of Au-DOX. f Size distribution and size in water of Au-DOX.** Fig. S3.** Characterization of PCL-PEOz- maleimide. a XPS spectra of PCL-PEOz- maleimide. b 1HNMR spectrum of the polymer.** Fig. S4**. Photographs of blank polymersomes (a-1) and cargo-loaded polymersomes (a-2). TEM images of blank polymersomes (b-1) and cargo-loading polymersomes (b-2).c In vitro toxicity evaluation of U87-MG cells after co-incubation with PO and ANG-PO for 24 h by Cell Counting Kit-8 (CCK-8) method.** Fig. S5.** The particle number of polymersomes was measured using NTA. The original sample was diluted 250 times before testing. The particle number of original sample was yielded a value of 6×1011 particles/mL after calculation.** Fig. S6**. Identification of primary astrocytes (a-1) and cerebral microvascular endothelial cells (a-2) by flow cytometry. b Expression levels of LRP1 on cell lines of U87-MG, BMECs, and normal astrocyte determined by Western blot. c Quantitative analysis of LRP1 protein levels. Data are presented as the mean plus or minus the standard deviation (SD), and n=3 for each group,**P<0.01,***P<0.001.** Fig. S7**. Confocal analysis of the U87 cells treated with Au-DOX@PO for 6 hours.

## Data Availability

The datasets used and analyzed during the current study are available from the corresponding author on reasonable request.
