# Supplementary material for: LRP1-mediated pH-sensitive polymersomes facilitate combination therapy of glioblastoma in vitro and in vivo
Source: J Nanobiotechnology. 2021 Jan 22;19:29. doi: 10.1186/s12951-020-00751-x (PMC7821499; doi:10.1186/s12951-020-00751-x)

**LRP1-mediated pH-sensitive polymersomes facilitate combination therapy of glioblastoma in vitro and in vivo**

Chen He^1^, Zhiyuan Zhang^2^, Yinan Ding^1^, Kangli Xue^1^, Xihui Wang^1^, Rui Yang^3^, Yanli An^1^, Dongfang Liu^1^, Chunmei Hu^4^* and Qiusha Tang^1^*

*^1^Medical School of Southeast University, 87 Dingjiaqiao Road, Nanjing, China.*

*^2^Department of Neurosurgery, Nanjing Jinling hospital, Nanjing University , Nanjing, China.*

*^3^Research Institute for Reproductive Health and Genetic Diseases, The Affiliated Wuxi Maternity and Child Health Care Hospital of Nanjing Medical University, Wuxi , China.*

*^4^Department of Tuberculosis, the Second Affiliated Hospital of Southeast University (the second hospital of Nanjing), Nanjing, China*

*Correspondence:

**Chunmei Hu and Qiusha Tang**

Department of Tuberculosis, the Second Affiliated Hospital of Southeast University (the second hospital of Nanjing), Nanjing, China

Medical School of Southeast University, 87 Dingjiaqiao Road, Nanjing, China.

hcm200702@163.com, panyixi-tqs@163.com

**Fig. S1.** Characterization of AuNPs, AuNPs-NH_2._ TEM images of AuNPs **(a-1)** and AuNPs-NH_2_ **(a-2)**. Size distribution and size in water of AuNPs **(b-1)** and AuNPs-NH_2_ **(b-2)**. **c** Zeta-potentials of AuNPs and AuNPs-NH_2_. **d** Spectrophotometer results of AuNPs and AuNPs-NH_2_. **e** In vitro toxicity evaluation of U87-MG cells after co-incubation with AuNPs-NH_2_ for 24 h by Cell Counting Kit-8 (CCK-8) method. **f** Dynamic light scattering (DLS) size measurements of AuNPs-NH_2_ in PBS for varied time durations (0 ~ 29 days).





**Fig. S2.** Characterization of Au-DOX. IC50 of DOX (**a**) and AuNPs combined with radiotherapy of 6Gy (**b**). **c** TEM image of Au-DOX. **d** Spectrophotometer results of AuNPs , AuNPs-NH2 and Au-DOX. **e** FT-IR spectrum of Au-DOX. **f** Size distribution and size in water of Au-DOX





**Fig. S3.** Characterization of PCL-PEOz- maleimide. **a** XPS spectra of PCL-PEOz- maleimide. **b** ^1^HNMR spectrum of the polymer.

**

**

**Fig. S4.** Photographs of blank polymersomes (**a-1**) and cargo-loaded polymersomes (**a-2**). TEM images of blank polymersomes (**b-1**) and cargo-loading polymersomes (**b-2**).**c** In vitro toxicity evaluation of U87-MG cells after co-incubation with PO and ANG-PO for 24 h by Cell Counting Kit-8 (CCK-8) method.





**Fig. S5.** The particle number of polymersomes was measured using NTA. The original sample was diluted 250 times before testing. The particle number of original sample was yielded a value of 6×10^11^ particles/mL after calculation.

**
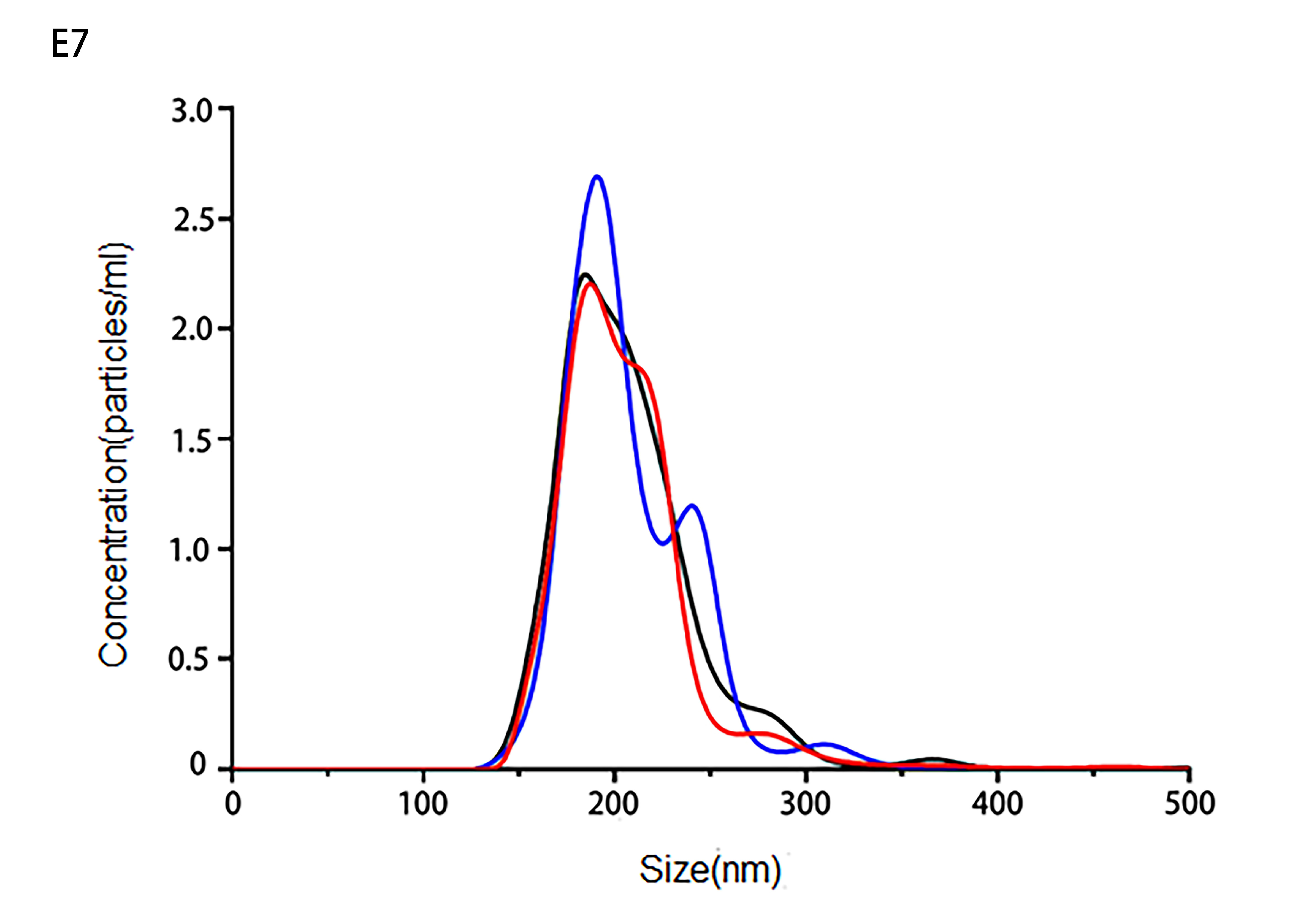
**

Stats: Mean +/- Standard Error:

Mean: 216.8 +/- 3.2 nm

Concentration (Upgrade): 2.40e+09 +/- 1.12e+08 particles/ml

**Fig. S6.** Identification of primary astrocytes (**a-1**) and cerebral microvascular endothelial cells (**a-2**) by flow cytometry. **b** Expression levels of LRP1 on cell lines of U87-MG, BMECs, and normal astrocyte determined by Western blot. **c** Quantitative analysis of LRP1 protein levels. Data are presented as the mean plus or minus the standard deviation (SD), and n=3 for each group,**P<0.01,***P<0.001.


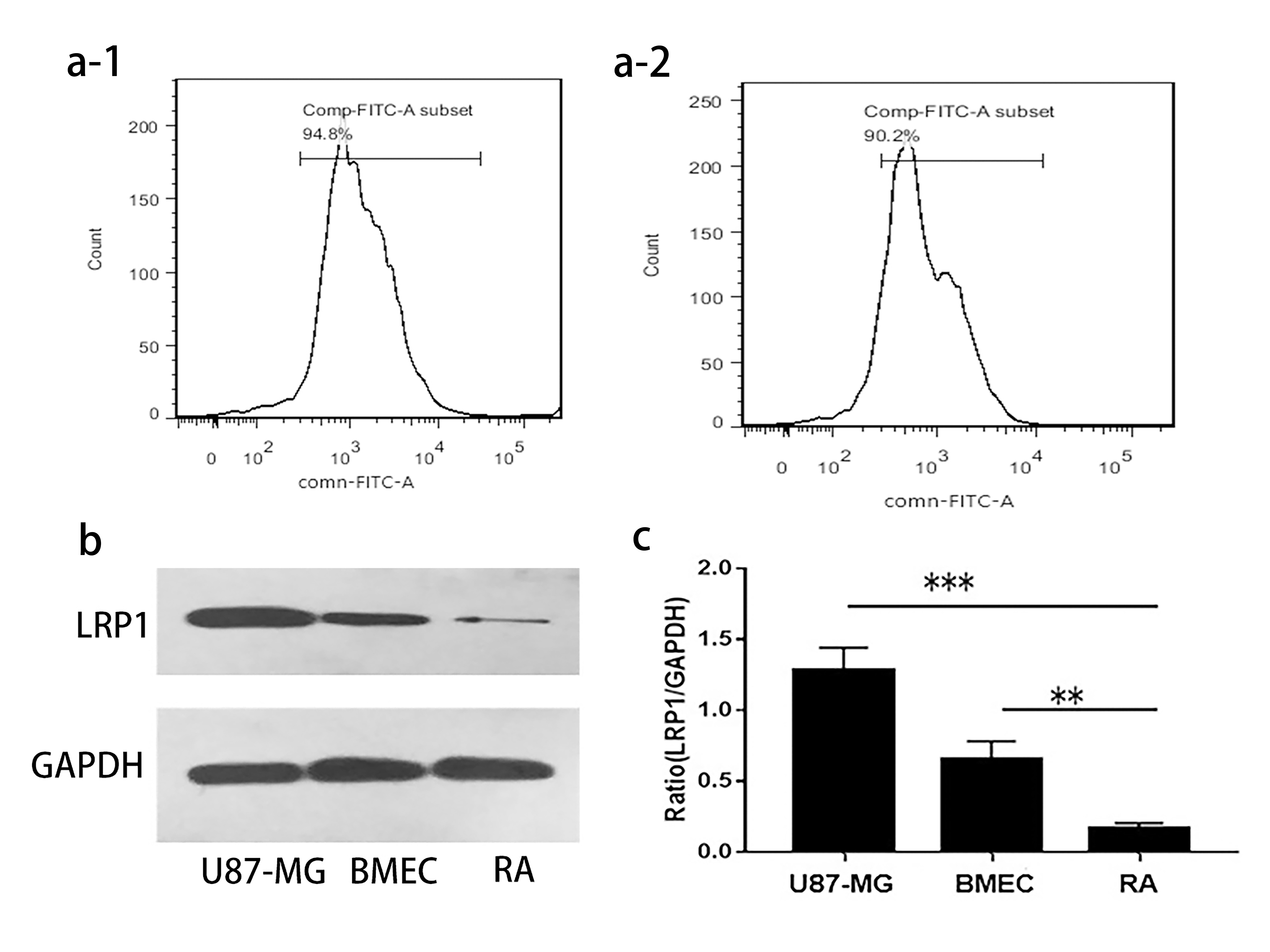


**Fig. S7.** Confocal analysis of the U87 cells treated with Au-DOX@PO for 6 hours.


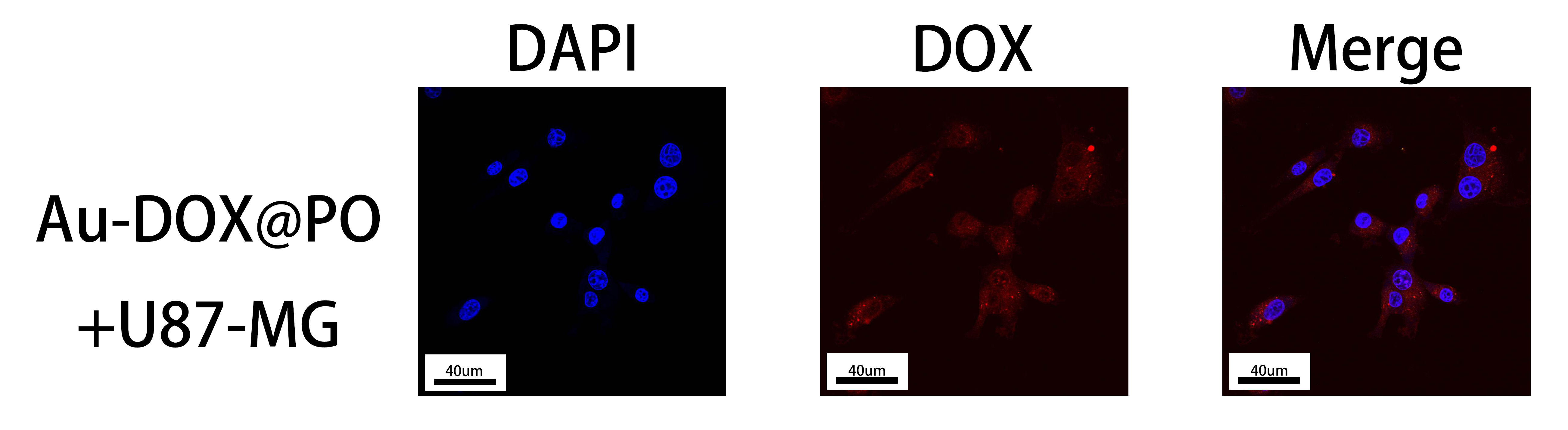

Supplement: Supplementary file 1 — Additional file 1: Fig. S1. Characterization of AuNPs, AuNPs-NH2. TEM images of AuNPs (a-1) and AuNPs-NH2 (a-2). Size distribution and size in water of AuNPs (b-1) and AuNPs-NH2 (b-2). c Zeta-potentials of AuNPs and AuNPs-NH2. d Spectrophotometer results of AuNPs and AuNPs-NH2. e In vitro toxicity evaluation of U87-MG cells after co-incubation with AuNPs-NH2 for 24 h by Cell Counting Kit-8 (CCK-8) method. f Dynamic light scattering (DLS) size measurements of AuNPs-NH2 in PBS for varied time durations (0 ~ 29 days). Fig. S2. Characterization of Au-DOX. IC50 of DOX (a) and AuNPs combined with radiotherapy of 6Gy (b). c TEM image of Au-DOX. d Spectrophotometer results of AuNPs , AuNPs-NH2 and Au-DOX. e FT-IR spectrum of Au-DOX. f Size distribution and size in water of Au-DOX. Fig. S3. Characterization of PCL-PEOz- maleimide. a XPS spectra of PCL-PEOz- maleimide. b 1HNMR spectrum of the polymer. Fig. S4. Photographs of blank polymersomes (a-1) and cargo-loaded polymersomes (a-2). TEM images of blank polymersomes (b-1) and cargo-loading polymersomes (b-2).c In vitro toxicity evaluation of U87-MG cells after co-incubation with PO and ANG-PO for 24 h by Cell Counting Kit-8 (CCK-8) method. Fig. S5. The particle number of polymersomes was measured using NTA. The original sample was diluted 250 times before testing. The particle number of original sample was yielded a value of 6×10^11^ particles/mL after calculation. Fig. S6. Identification of primary astrocytes (a-1) and cerebral microvascular endothelial cells (a-2) by flow cytometry. b Expression levels of LRP1 on cell lines of U87-MG, BMECs, and normal astrocyte determined by Western blot. c Quantitative analysis of LRP1 protein levels. Data are presented as the mean plus or minus the standard deviation (SD), and n=3 for each group,**P<0.01,***P<0.001. Fig. S7. Confocal analysis of the U87 cells treated with Au-DOX@PO for 6 hours. [file 12951_2020_751_MOESM1_ESM.docx]
